# Supplementary material for: Evaluation of Polyphenolic Composition and Antimicrobial Properties of Sanguisorba officinalis L. and Sanguisorba minor Scop
Source: Plants (Basel). 2022 Dec 16;11(24):3561. doi: 10.3390/plants11243561 (PMC9785539; doi:10.3390/plants11243561)
Supplement: Supplementary file 1 [file plants-11-03561-s001.zip › plants-2098448-supplementary.pdf]

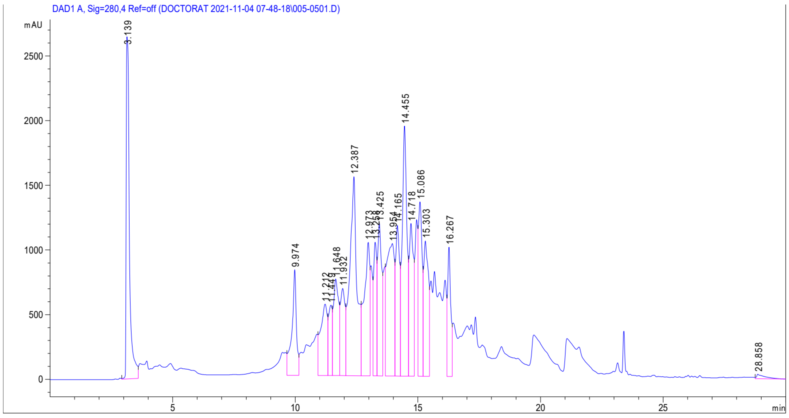

a-SOR

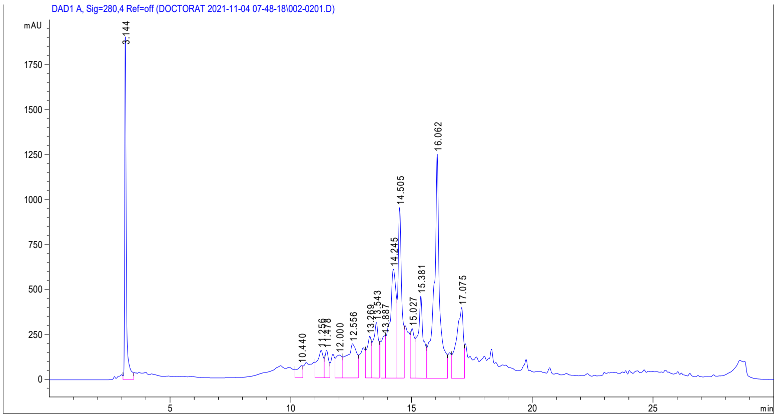

b-SOL

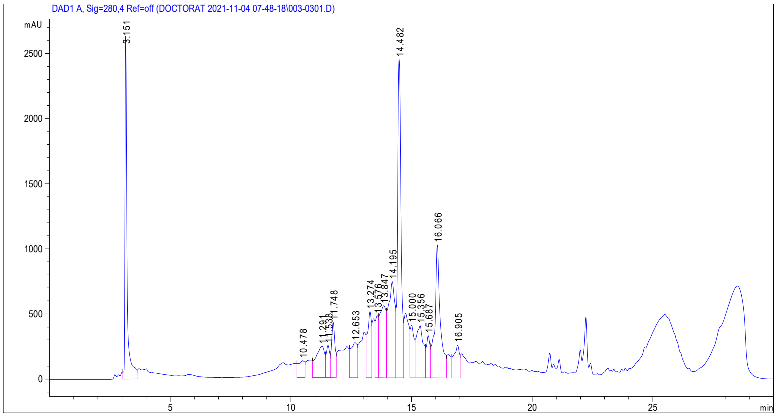

c-SOF

Figure S1. HPLC cromatograms of *Sanguisorba officinalis* L., a. *SOR*-*S. officinalis* roots; b. SOL-*S. officinalis* leaves, c. SOF-*S. officinalis* flowers

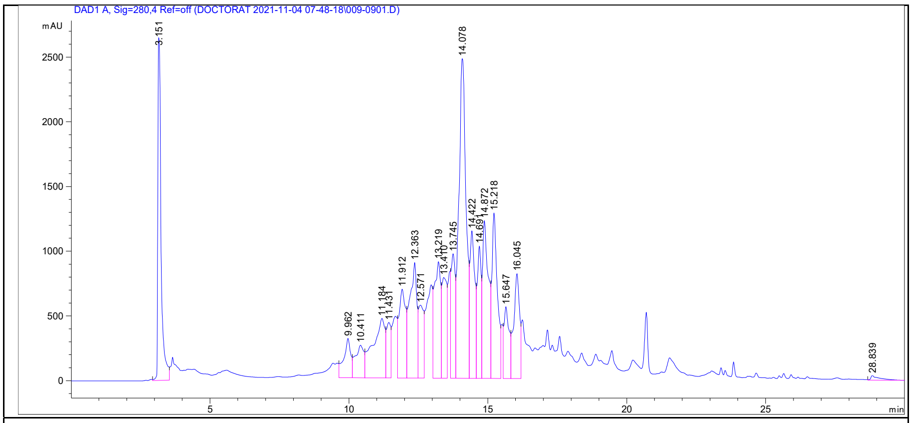

a-SMR

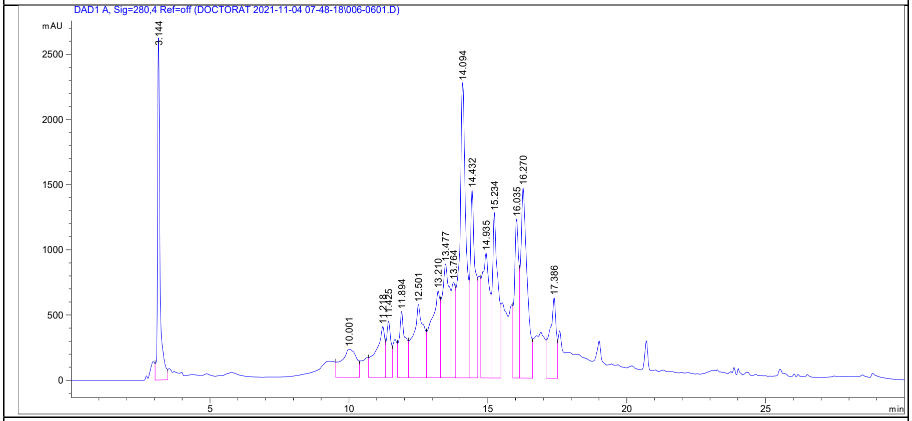

b-SML

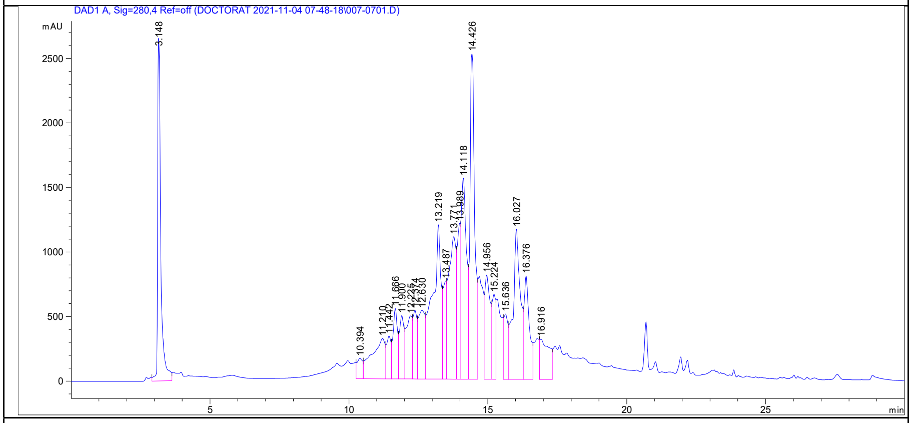

c-SMF

Figure S2. HPLC Cromatograms of *Sanguisorba minor* Scop., a.SMR-*S. minor* roots; b. SML-*S. minor* leaves; c. SMF-*S. minor* flowers

Table S1. Antimicrobial activity of antibiotics against gram (+) (*S.aureus*) and gram (-) bacteria strains (*E. coli* and *P. aeruginosa*) expressed as diameter of inhibition zone.

Bacteria strains / Antibiotics	*E.coli*	*P. aeruginosa*	*S. aureus*
	Diameter of inhibition zone (mm)*		
Azithromycin	-	-	21.00 ± 0.00
Ciprofloxacin	35.03 ± 0.06	25.5 ± 0.5	25.66 ± 0.58
Cefoxitin	-	-	29.00 ± 0.00
Clindamycin	-	-	29.00 ± 0.00
Ceftriaxone	34.00 ± 0.00	15.33 ± 0.58	-
Doxycycline	-	-	25.16 ± 0.29
Gentamycin	24.07 ± 0.12	15.00 ± 0.00	-
Meropenem	30.66 ± 0.57	15.66 ± 0.58	-
Nitrofurantoin	23.00 ± 0.00	-	-

Data are expressed as the mean value ±SD (n = 3). The "-" sign means that no antimicrobial activity was observed.
